# Effect of Various Nanofillers on Piezoelectric Nanogenerator Performance of P(VDF-TrFE) Nanocomposite Thin Film

**DOI:** 10.3390/nano15050403

**Published:** 2025-03-06

**Authors:** Hafiz Muhammad Abid Yaseen, Sangkwon Park

**Affiliations:** Department of Chemical and Biochemical Engineering, Dongguk University, 30 Pildong-ro 1-gil, Jung-gu, Seoul 04620, Republic of Korea

**Keywords:** piezoelectric nanogenerator (PENG), nanofillers, P(VDF-TrFE) nanocomposite thin film, PENG performance

## Abstract

Flexible polymer-based piezoelectric nanogenerators (PENGs) have gained significant interest due to their ability to deliver clean and sustainable energy for self-powered electronics and wearable devices. Recently, the incorporation of fillers into the ferroelectric polymer matrix has been used to improve the relatively low piezoelectric properties of polymer-based PENGs. In this study, we investigated the effect of various nanofillers such as titania (TiO_2_), zinc oxide (ZnO), reduced graphene oxide (rGO), and lead zirconate titanate (PZT) on the PENG performance of the nanocomposite thin films containing the nanofillers in poly(vinylidene fluoride-co-trifluoro ethylene) (P(VDF-TrFE)) matrix. The nanocomposite films were prepared by depositing molecularly thin films of P(VDF-TrFE) and nanofiller nanoparticles (NPs) spread at the air/water interface onto the indium tin oxide-coated polyethylene terephthalate (ITO-PET) substrate, and they were characterized by measuring their microstructures, crystallinity, β-phase contents, and piezoelectric coefficients (*d*_33_) using SEM, FT-IR, XRD, and quasi-static meter, respectively. Multiple PENGs incorporating various nanofillers within the polymer matrix were developed by assembling thin film-coated substrates into a sandwich-like structure. Their piezoelectric properties, such as open-circuit output voltage (*V_OC_*) and short-circuit current (*I_SC_*), were analyzed. As a result, the PENG containing 4 wt% PZT, which was named P-PZT-4, showed the best performance of *V_OC_* of 68.5 V with the *d*_33_ value of 78.2 pC/N and β-phase content of 97%. The order of the maximum *V_OC_* values for the PENGs of nanocomposite thin films containing various nanofillers was PZT (68.5 V) > rGO (64.0 V) > ZnO (50.9 V) > TiO_2_ (48.1 V). When the best optimum PENG was integrated into a simple circuit comprising rectifiers and a capacitor, it demonstrated an excellent two-dimensional power density of 20.6 μW/cm^2^ and an energy storage capacity of 531.4 μJ within 3 min. This piezoelectric performance of PENG with the optimized nanofiller type and content was found to be superior when it was compared with those in the literature. This PENG comprising nanocomposite thin film with optimized nanofiller type and content shows a potential application for a power source for low-powered electronics such as wearable devices.

## 1. Introduction

In modern times, with the development of new and advanced electrical devices across various fields, efficient electrical energy generation has become a significant and practical theme in engineering research [1]. Energy-harvesting nanogenerators (NGs) are emerging as reliable alternatives to batteries in low-power wireless electronic devices, sparking significant research interest in this modern trend. Ambient motions serve as a primary source for energy harvesting, and numerous motion-based energy harvesters have been demonstrated on the microscale level [2]. Notably, the human body generates several kilowatts of energy through daily activities, making biomechanical energy harvesting, self-powered systems, and wearable electronics a focal point of research. This has led to the development of piezoelectric nanogenerators (PENGs) using various piezoelectric materials [3].

Recently, among these piezoelectric materials, poly(vinylidene fluoride-co-trifluoroethylene) (P(VDF-TrFE)) has gained much attention due to its high piezoelectric coefficient, flexibility, and ease of processing [4]. This semicrystalline polymer exhibits both polar and nonpolar crystalline phases (α, β, γ, δ, and ε) depending on its crystallization conditions. While the α-phase is nonpolar, the polar β-phase possesses exceptional piezoelectric properties, making it highly desirable. To induce the β-phase by aligning the dipoles in the polymer, techniques such as the addition of nanofillers, thermal annealing, mechanical stretching, or electric poling are commonly employed [5,6]. Nanofillers, including carbon-based materials, piezoelectric ceramics, and metal oxides, have also been extensively used to enhance the β-phase content and overall performance of PVDF and its copolymers [4,7,8].

Metal oxide nanofillers, such as TiO_2_ and ZnO, are intensively studied due to their widespread applications in electronic and piezoelectric systems [9,10,11,12,13]. TiO_2_ offers excellent chemical and thermal stability, photostability, and high photoconversion efficiency [10], though its piezoelectric coefficient is lower than that of ZnO. ZnO nanostructures have attracted attention for their unique properties, including non-toxicity, biological compatibility, versatile conductivity, ferroelectric and ferromagnetic behaviors, wide bandgap semiconducting properties, and a strong piezoelectric response [12]. On the other hand, carbon-based nanofillers including carbon nanotube (CNT), carbon black, graphene (Gr), graphene oxide (GO), and reduced graphene oxide (rGO), with superior electrical conductivity and high surface area, have been known to be promising candidates [14,15]. Meanwhile, piezoceramic materials including barium titanate (BaTiO_3_, BTO) and lead zirconate titanate (PbZr_x_Ti_1−x_O_3_, PZT) have been reported to be desirable as nanofillers because they have exceptional piezoelectric properties, including a higher piezoelectric strain constant (*d*_33_), compared to other nanofillers, and minimal mechanical losses [16,17,18,19,20].

These nanofillers are known to act as nucleating agents, enhancing the crystallinity and the β-phase content of PVDF-based nanocomposites; thus, selecting proper fillers with high piezoelectric constants may increase piezoelectricity [21,22]. However, excessive filler content in PVDF matrices can reduce flexibility and piezoelectricity, presumably due to problems such as aggregation and difficulty in uniform dispersion. Therefore, optimizing filler content is crucial for enhanced piezoelectric performance [23]. In other words, selecting optimized nanofiller content and type is essential to fabricating efficient nanocomposite-based PENGs.

In this work, for the first time, we conducted a comparative study on the performance of PENGs based on nanocomposite thin films comprising various nanofillers dispersed in the piezoelectric P(VDF-TrFE) matrix. These nanofillers include TiO_2_, ZnO, rGO, and PZT, representing classes of metal oxides, carbon-based materials, and piezoelectric ceramics, respectively. We utilized the Langmuir–Schaefer (LS) technique to fabricate nanocomposite thin films [15], as it is an excellent method for depositing uniform and highly ordered thin films on solid substrates [24] and providing a high β-phase content by aligning dipoles in its preparation procedure [25]. In addition, when a PENG is fabricated based on the LS thin films, enhanced piezoelectric performance is anticipated because one can control thin film preparation parameters such as surface pressure, filler content and type, and the number of layers (film thickness). Since this study focused on comparing the piezoelectric performance of nanocomposite thin films with various nanofillers, representing important classes of materials at different nanofiller contents, the results were intended to reveal what nanofiller content is optimum and which nanofiller classes show better piezoelectric behavior in nanocomposite thin films of P(VDF-TrFE).

## 2. Materials and Methods

### 2.1. Materials

P(VDF-TrFE) (70:30 mol%), titanium dioxide nanopowder (TiO_2_, average particle size 21 nm), zinc oxide (ZnO, average particle size < 100 nm), reduced graphene oxide (rGO, surface area 103 m^3^/g), acetone (99.7%), *N*,*N*-dimethylformamide (DMF, 99.8%), ethanol (96%), and indium tin oxide-coated polyethylene terephthalate (ITO-PET) were obtained from the Merck Korea, Seoul, Republic of Korea, Lead zirconate titanate powder (PZT, average particle size 100–150 nm) was supplied by Nanoshel Korea Limited, Seongnam, Republic of Korea. All the chemicals and materials were used without any further purification. Deionized (DI) water with a resistivity of 18.3 MΩ·cm was used.

### 2.2. Preparation of Nanocomposite Solution and Thin Film

**Figure 1:**
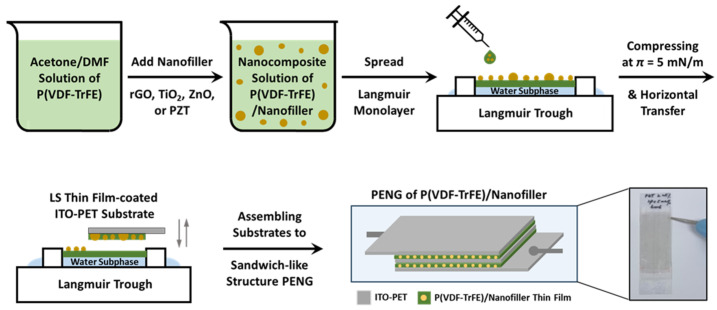
Illustration of preparation procedure of nanocomposite LS film and their PENG device.

Nanocomposite solutions were prepared by dissolving 125 mg of P(VDF-TrFE) and dispersing specific amounts of nanofiller in 250 g of an acetone/DMF mixture (*v*/*v* 6:4). The nanofiller amounts used were 2.5, 5, 25, and 50 mg, corresponding to 2, 4, 20, and 40 wt% relative to the P(VDF-TrFE) weight, and 0.001, 0.002, 0.01, and 0.02 wt% relative to the spreading solution weight, respectively. For TiO_2_-based nanocomposites, these solutions were labeled as P-TiO_2_-2, P-TiO_2_-4, P-TiO_2_-20, and P-TiO_2_-40, respectively, for ZnO, P-ZnO-2, P-ZnO-4, P-ZnO-20, and P-ZnO-40, respectively, for rGO, P-rGO-2, P-rGO-4, P-rGO-20, and P-rGO-40, respectively, and for PZT, P-PZT-2, P-PZT-4, P-PZT-20, and P-PZT-40, respectively. Pristine P(VDF-TrFE) was named P, P-TiO_2_-0, P-ZnO-0, P-rGO-0, or P-PZT-0, because it has no nanofillers. Nanocomposite thin films composed of P(VDF-TrFE) and nanofiller particles were fabricated using the Langmuir–Schaefer (LS) technique. Initially, an appropriate amount (typically 500 μL) of nanocomposite solution was spread onto the surface of double-distilled water in a clean Teflon Langmuir trough using a micro-syringe. The spread monolayer was then compressed to a surface pressure of 5 mN/m by moving barriers at a speed of 10 mm/min. Next, the monolayer was transferred onto an ITO-PET substrate by horizontally touching it with the substrate. A six-layer LS film was obtained by repeating this transfer process six times. Finally, all films were dried at ambient temperature (23–25 °C) and stored securely under vacuum for further analysis. The PENG fabrication procedure is illustrated in Figure 1.

### 2.3. Characterization of Nanocomposite Thin Film

The microstructure and morphology of the P(VDF-TrFE)/nanofiller nanocomposite LS films were examined using a scanning electron microscope (SEM, JSM-6700 F, Jeol Ltd., Tokyo, Japan). Their crystalline structures were analyzed with an X-ray diffractometer (XRD, D/Max2500, Rigaku, Tokyo, Japan), while FT-IR spectroscopy (Nicolet iS50, Thermo Fisher Scientific, Waltham, MA, USA) was used to assess the crystalline phases within the 500–4000 cm^−1^ range. The piezoelectric coefficient (*d*_33_) was determined using a quasistatic meter (Han Tech, PM 3500, Seoul, Republic of Korea) with a force of 0.25 N at a frequency of 110 Hz and a force head dimension of 110 mm × 140 mm after the calibration with a standard sample.

### 2.4. Fabrication of PENGs and Measurement of Nanogenerator Performance

To fabricate a PENG, the two LS film-coated ITO-PET sheets were assembled as a sandwich-like structure where the film-coated areas (of about 6 cm × 2.5 cm) were arranged to face each other, and two outsides of the sandwiched plates were covered with new pristine ITO-PETs, respectively. The uncoated ITO parts (about 2 cm × 2.5 cm) were placed on opposite sides to connect electrodes to a digital storage oscilloscope (EDUX1002G, Keysight Technologies, Santa Rosa, CA, USA) with alligators. Figure 1 illustrates a representative fabricated PENG and its sandwich-like structure. To evaluate the piezoelectric performance of the PENG device, the open-circuit voltage (*V_OC_*) and the short-circuit current (*I_SC_*) were measured. The device was initially mounted on a motorized Ezi-Servo bending machine (Fastech, EzS-NDR-42, Bucheon, Republic of Korea). Subsequently, the device was exposed to constant strain through bending and releasing motions with a constant bending angle of 50° at 1 Hz and 2.5 Hz frequencies. During this process, the *V_OC_* and the *I_SC_* were simultaneously measured using a digital oscilloscope.

## 3. Results and Discussion

### 3.1. Microstructure and Morphology

**Figure 2:**
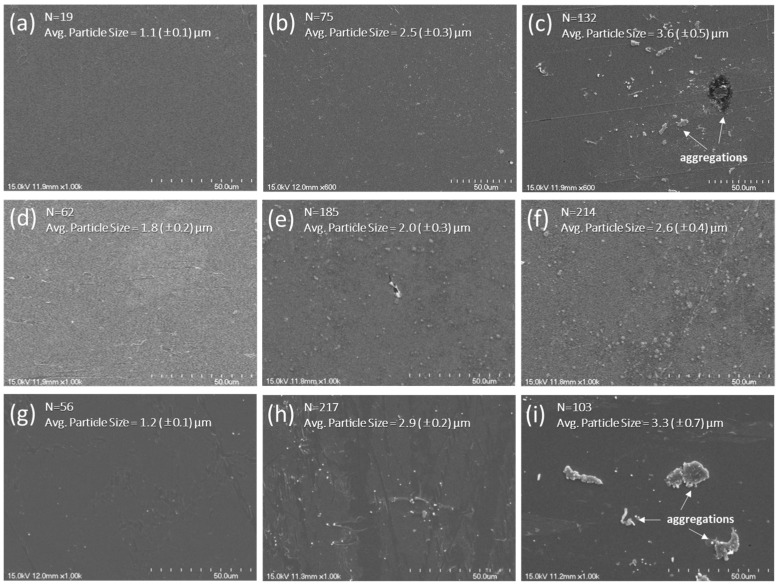
SEM micrographs of nanocomposite LS thin films of (**a**) P, (**b**) P-TiO_2_-4, (**c**) P-TiO_2_-40, (**d**) P-ZnO-2, (**e**) P-ZnO-20, (**f**) P-ZnO-40, (**g**) P-PZT-2, (**h**) P-PZT-20, and (**i**) P-PZT-40 with 50 μm scale bars.

The microstructure and morphology of nanocomposite thin films were observed using a SEM. Figure 2 shows the images for the thin film samples of P, P-TiO_2_-4, P-TiO_2_-40, P-ZnO-2, P-ZnO-20, P-ZnO-40, P-PZT-2, P-PZT-20, and P-PZT-40. As seen in Figure 2b,d,g, the thin films with 2 and 4 wt% of nanofillers showed uniform and homogeneous microstructures with a small number of aggregates of 1.1–2.5 μm average particle sizes, regardless of nanofiller type. Considering that the sizes of nanofiller primary particles were about 21 nm, less than 100 nm, and 100–150 nm for TiO_2_, ZnO, and PZT, respectively, this indicates that most of the nanofiller primary particles were uniformly distributed in most parts of the thin films up to 4 wt% of nanofiller content. When the thin films contained 20 wt% of nanofiller, their uniformity and homogeneity slightly decreased with larger aggregates of 2.0–2.9 μm, as seen in Figure 2e,h. Up to 20 wt% of nanofiller, most aggregates appeared spherical. As seen in Figure 2c,f,i, when the thin films contained 40 wt% nanofillers, their uniformity and homogeneity became worse with some larger aggregates (especially larger than 10 μm for PZT) of various shapes. Considering nanofiller primary particle*s’* sizes, these aggregates were agglomerations of a few tens to hundreds of primary particles. Achieving a homogeneous dispersion of nanofillers in the polymer matrix is crucial for piezoelectric applications because it enables uniform stress distribution across the nanocomposite thin films [26,27,28]. This film uniformity and homogeneity would result in even polarization when pressure is applied, which enhances the efficiency of transforming mechanical energy into electricity [26,27,28]. Since the thickness of the polymer matrix (six layers of P(VDF-TrFE) LS film) used in this study was estimated to be about 24 nm [15], the large aggregates observed in the thin films containing a higher content of nanofiller were presumed to form protruding structures, like islands, which not only hamper even polarization but also negatively affect the electrical contact and mechanical characteristics of the films in PENG devices. Thus, these SEM observation results implied that the thin films containing fewer nanofillers, such as 2 and 4 wt%, show better piezoelectric properties than those with higher content, such as 20 and 40 wt%.

### 3.2. Crystallinity and β-Phase Content

**Figure 3:**
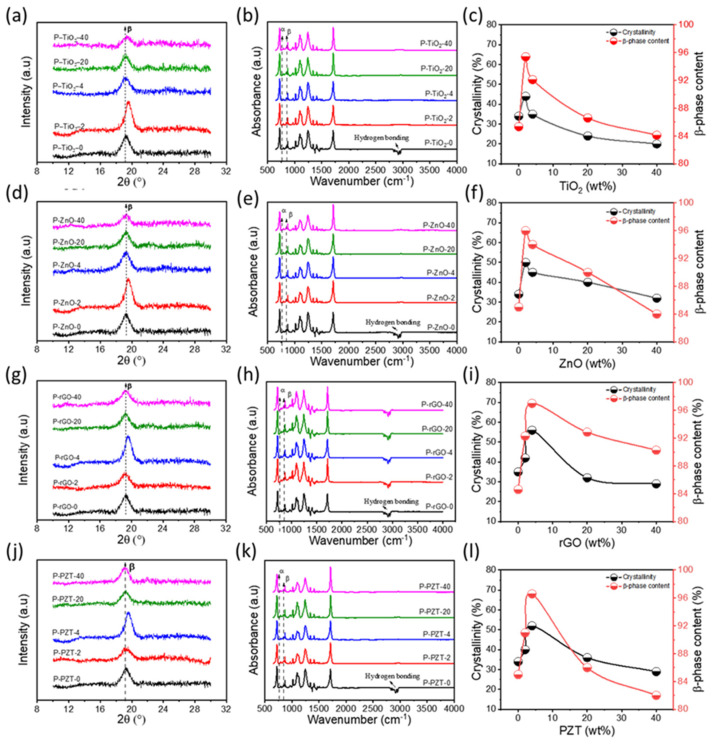
XRD patterns, FT-IR spectra, crystallinity, and β-phase content profiles as a function of nanofiller content for the nanocomposite thin films of (**a**–**c**) P-TiO_2_, (**d**–**f**) P-ZnO, (**g**–**i**) P-rGO, and (**j**–**l**) P-PZT series.

Crystallinity can be determined using specific diffraction angles (2θ) and intensity data from an XRD diffraction pattern, which reflect the interplanar orientation and the amount of the corresponding crystal plane. Figure 3a,d,g,j present the XRD patterns of P-TiO_2_, P-ZnO, P-rGO, and P-PZT nanocomposite thin films at different nanofiller content, respectively. As shown in those Figures, all the thin films yielded peaks at about 2θ = 19.2–19.8°, which correspond to (100) and (200) planes of β-phase [29]. From the peak area, the percentage crystallinity was calculated by the following equation [30]:

(1) $$X_{C}\;\left(\%\right)=\frac{\sum \;A_{Crys}}{\sum A_{Crys}+\sum A_{Amor}}\times 100$$

where ∑*A_Crys_* and ∑*A_Amor_* denote the total area under crystalline and amorphous regions, respectively. For the pristine P(VDF-TrFE) film, the percentage of crystallinity was 34%. As shown in Figure 3c,f,i,l, all the thin films showed a similar trend: the crystallinity initially increased as nanofiller content increased, reached a maximum at 2 or 4 wt% nanofiller content, and then decreased. The maximum crystallinity values were 44%, 50%, 56%, and 52% for the samples of P-TiO_2_-2, P-ZnO-2, P-rGO-4, and P-PZT-4, respectively. This maximum crystallinity behavior at a specific nanofiller content explains that nanofiller NPs play the role of crystallization nuclei and keep promoting crystallization up to 2–4 wt% content. However, more nanofillers start to hamper polymer molecules from crystallizing by restricting polymer chain mobility due to the formation of aggregations (as in Figure 2c,f,i) [31,32].

As shown in the FT-IR spectra in Figure 3b,e,h,k, all the films exhibited sharp peaks at several wavelengths. The peaks at 847, 874, 1177, and 1410 cm^−1^ corresponded to the β-phase peaks, whereas those at 761, 971, and 1119 cm^−1^ resulted from α-phases [25]. Among them, the α-phase peak at 761 cm^−1^ and the β-phase peak at 847 cm^−1^ were utilized to determine the β-phase content (%) using the following equation [33,34]:

(2) $$F\left(\beta \right)=\frac{\;A_{\beta }}{1.26\;A_{\alpha }+\;A_{\beta }}\times 100$$

where *A_α_* and *A_β_* are the absorbance values at 761 and 847 cm^−1^, respectively. As shown in Figure 3c,f,i,l, for all the films, there is a clear trend: the β-phase content (*F*(*β*)) of 85% for the pristine P(VDF-TrFE) thin film initially increased as nanofiller content increased, reached maximum values (95% for P-TiO_2_-2, 96% for P-ZnO-2, 97% for P-rGO-4, and 97% for P-PZT-4) at 2 or 4 wt% nanofiller content, and then continued to decrease to lower value. The crystallinity and the β-phase content results are shown in Table 1.

### 3.3. Piezoelectric Coefficient

The piezoelectric coefficient (*d*_33_) of the nanocomposite thin film is a key factor in influencing the open-circuit output voltage (*V_OC_*) and short-circuit current (*I_SC_*) of a PENG, as demonstrated by the following equations: [35,36]:

(3) $$I_{SC}=d_{33}YAe$$

(4) $$V_{OC}=g_{33}\sigma Yt$$

In these equations, *Y*, *A*, and *e* represent Young’s modulus, the cross-sectional area, and the strain, respectively. The symbols *σ* and *t* denote the strain in the perpendicular direction and the device thickness, respectively. The piezoelectric voltage constant, *g*_33_, is defined as *g*_33_ = *d*_33_/(*ε*_0_*ε*), where *ε*_0_ is the dielectric constant of free space and ε is the dielectric constant of the nanocomposite material.

The dielectric constant *ε* is expressed as the following equation [37]:

(5) $$\varepsilon =\left(C\;d\right)/\left(\varepsilon_{0}A\right)$$

where *C* and *d* are the capacitance and the nanocomposite thin film thickness. Therefore, the relationship of *g*_33_ = *d*_33_ (*A*/(*C d*)) and the following equation hold:

(6) $$V_{OC}=d_{33}\left(\frac{\sigma AYt}{C\;d}\right)$$

Consequently, both *I_SC_* and *V_OC_* are proportional to *d*_33_, assuming that physical and mechanical parameters, such as *Y*, *σ*, *A*, *t*, *d*, and *C*, are constant.

**Figure 4:**
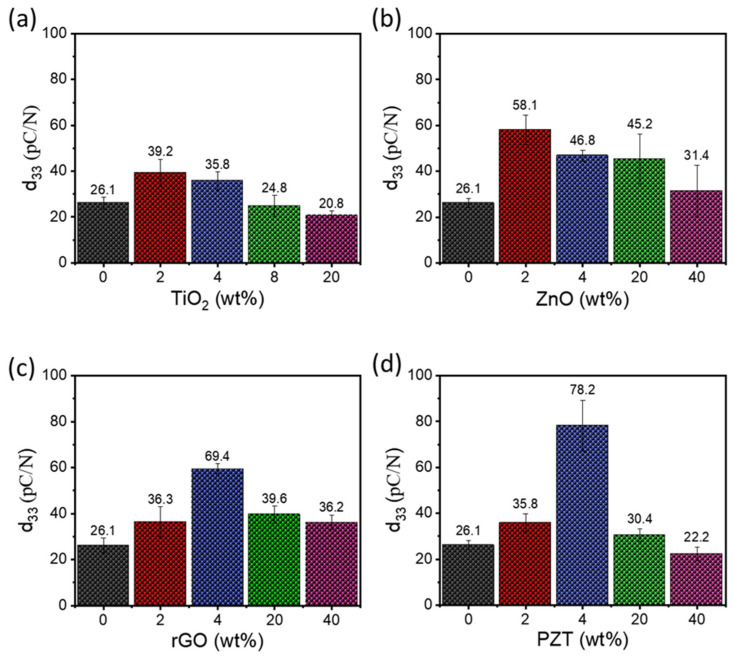
Piezoelectric coefficient (*d*_33_) values as a function of nanofiller content for the nanocomposite thin films of (**a**) P-TiO_2_, (**b**) P-ZnO, (**c**) P-rGO, and (**d**) P-PZT series.

The piezoelectric coefficient (*d*_33_) was measured by the quasi-static meter at room temperature and a constant force of 0.25 N at 110 Hz. The results are shown in Figure 4 and summarized in Table 2.

As seen in Figure 4 and Table 2, there were distinct trends of *d*_33_ for all the films: the *d*_33_ value initially increased as nanofiller content increased, reached a maximum at 2 or 4 wt% of nanofiller content, and then decreased. The maximum *d*_33_ values were 39.2, 58.1, 69.4, and 78.2 pC/N for the P-TiO_2_-2, P-ZnO-2, P-rGO-4, and P-PZT-4, respectively. In particular, the maximum values were in the order of P-PZT-4 > P-rGO-4 > P-ZnO-2 > P-TiO_2_-2, and those for P-PZT-4 and P-rGO-4 were about 1.77 and 1.99 times higher than that for P-TiO_2_-2. Considering that the *d*_33_ values for pure P(VDF-TrFE) and PZT are 25–40 and 225–590 pC/N, respectively [38], the highest *d*_33_ value of 78.2 pC/N for the P-PZT-4 nanocomposite film is reasonable. However, this value of 78.2 pC/N was about 65% higher than the arithmetic mean value of 47.5 pC/N. This implies that there may be a synergistic effect for PZT nanofiller in the P(VDF-TrFE) matrix. It is explained that at the optimum PZT content, the highly poled PZT NPs generate an internal field favoring the orientation of the polymer molecules, whereas the PZT nanoparticles hamper the orientation due to the formation of nonuniform aggregation at the higher PZT content [39,40].

### 3.4. Energy Harvesting

#### 3.4.1. Piezoelectric Performance

**Figure 5:**
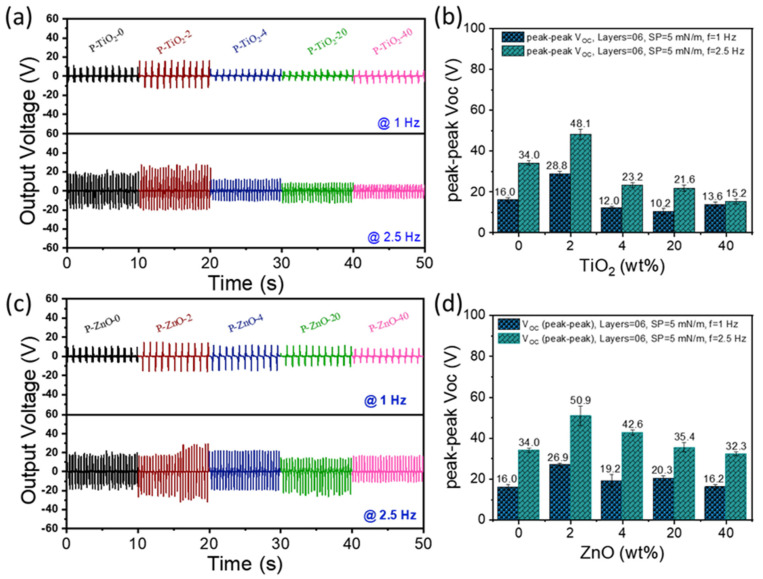

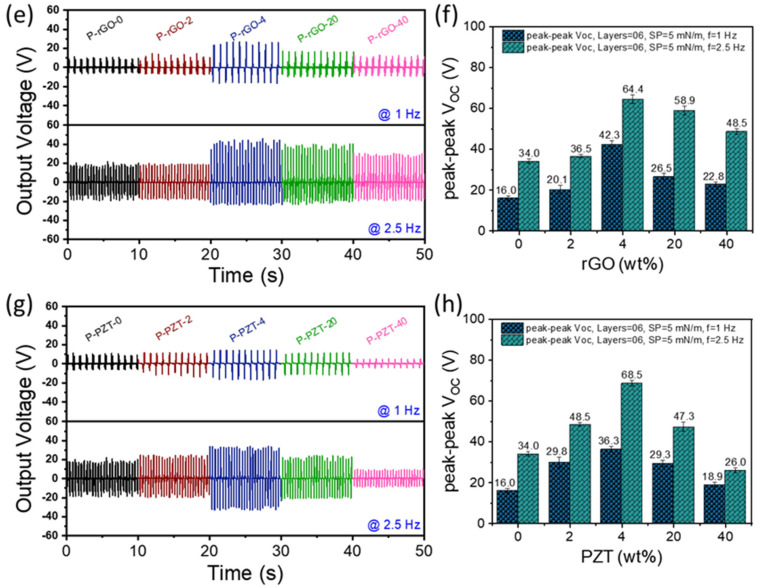
*V_OC_* signals of PENG devices and maximum peak-to-peak *V_OC_* values as a function of nanofiller content for the nanocomposite thin films of (**a**,**b**) P-TiO_2_, (**c**,**d**) P-ZnO, (**e**,**f**) P-rGO, and (**g**,**h**) P-PZT series.

The P(VDF-TrFE)/nanofiller nanocomposite thin films, incorporating four different types of nanofillers at varying contents of 0, 2, 4, 20, and 40 wt%, were utilized to fabricate sixteen distinct PENG devices, as illustrated in Figure 1. These PENG devices underwent bending–releasing tests at 1 and 2.5 Hz, during which the open-circuit output voltage (*V_OC_*) and short-circuit current (*I_SC_*) were measured using an oscilloscope. During cyclic bending and releasing, the PENGs generated piezoelectric potential. This occurs as the compressive deformation dissipates upon releasing the bending force, causing the generated electrons to flow back in an attempt to restore the original potential. This flow results in alternating positive and negative output voltages. Figure 5a,h depict the *V_OC_* signals for these PENGs, along with the maximum peak-to-peak *V_OC_* values as a function of filler content. Two key trends were identified in Figure 5. First, the peak-to-peak *V_OC_* values at 2.5 Hz were consistently higher than those at 1.0 Hz, with an increase ranging from 1.2 to 2.2 times. This can be attributed to the linear proportionality between the piezoelectric coefficient (*d*_33_) and the logarithm of the mechanical excitation frequency, making the *V_OC_* directly proportional to the frequency of mechanical deformation [41,42]. Second, as the nanofiller content increased, the peak-to-peak *V_OC_* initially rose, reached a maximum, and then declined. For instance, at 2.5 Hz, the PENG containing PZT exhibited a peak-to-peak *V_OC_* of 48.5 V at 2 wt% PZT content, which increased to a maximum of 68.5 V at 4 wt% PZT content before decreasing with further nanofiller addition. This maximum peak-to-peak *V_OC_* at 4 wt% PZT content can be attributed to the combined effects of crystallinity, β-phase content, and *d*_33_ results. In other words, the *V_OC_* was maximum at 4 wt% content because all the crystallinity, β-phase content, and *d*_33_ reached maximum values at the same content. The PENGs containing other nanofillers also showed a similar trend for the same reason. The composite of ferroelectric polymer containing ceramic filler particles may have hybrid characteristics of piezoelectric nanogenerator (PENG) and triboelectric nanogenerator (TENG) under the influence of an external force [18], and the TENG contribution is usually measured to be larger than the PENG [43]. In this study, the nanocomposite thin films of the P(VDF-TrFE) and nanofiller NPs might have similar hybrid characteristics because of their sandwich-like structure, and it should be noted that the TENG effect, as well as the PENG, might significantly contribute to the measured *V_OC_*.

**Figure 6:**
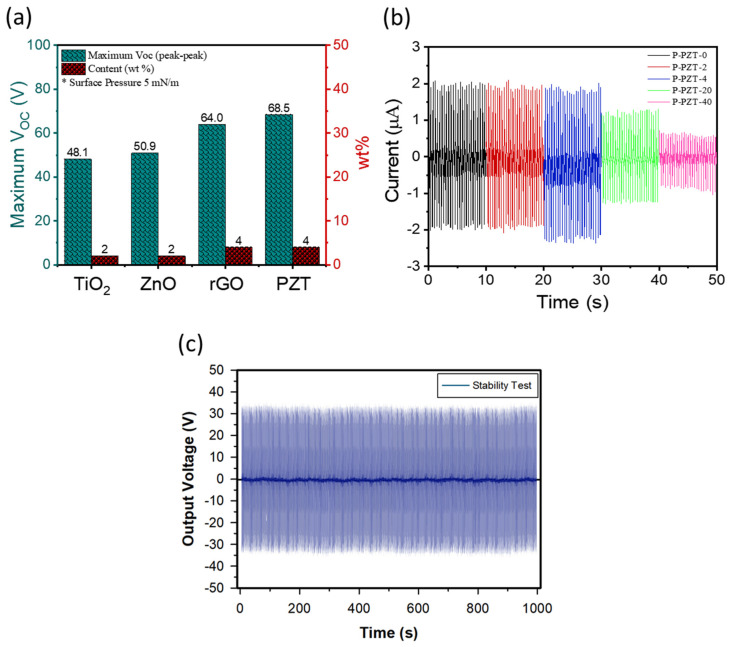
(**a**) The maximum *V_OC_* values for different optimum PENGs containing different nanofillers (with the surface pressure of 5 mN/m), (**b**) *I_SC_* signals for the PZT PENGs as a function of nanofiller content, and (**c**) stability of the optimized PZT-based PENG for 1000 s.

The PENG with the maximum peak-to-peak *V_OC_* is called the ‘optimum’ or the ‘optimal’ PENG hereafter. As shown in Figure 6a, the maximum *V_OC_* value was the highest for the PENG of P-PZT-4 and was in the order of P-PZT-4 (68.5 V) > P-rGO-4 (64.0 V) > P-ZnO-2 (50.9 V) > P-TiO_2_-2 (48.1 V), which was same as those for the three properties of crystallinity, β-phase content, and *d*_33_. The *I_SC_* values for the PZT PENGs were also measured, and the results are shown in Figure 6b. Similar to the *V_OC_* results, the PENG containing 4 wt% PZT exhibited the highest peak-to-peak *I_SC_* value of 4.4 μA, slightly surpassing the 4.0 μA observed for the pristine P(VDF-TrFE) film. From the measured values of *V_OC_*, *I_SC_*, the active area (*A_a_*), and the film thickness (*d*), the current density (*J_SC_*), the two-dimensional power density (*P*_2*D*_), and the three-dimensional power density (*P*_3*D*_) were calculated using equations of $J_{SC}=I_{SC}/A_{a}$ , $P_{2D}=V_{OC}I_{SC}/A_{a}$ , and $P_{3D}=V_{OC}I_{SC}/(A_{a}d)$ . The calculated values for the PENGs comprising P-PZT-0 and P-PZT-4 films are compared in Table 3. The *P*_2*D*_ for the optimal PENG of P-PZT-4 film was 20.6 μW/cm^2^, corresponding to the *P*_3*D*_ value of 8.6 W/cm^3^, assuming the film thickness of 24 nm. These values are about 2.3 times higher than those for the pristine-P(VDF-TrFE) film, P-PZT-0. Importantly, this *P*_2*D*_ value for the optimum PENG was about five times higher than a typical power density value of 4 μW/cm^2^, which is harvestable from solar energy [3]. Therefore, these results demonstrate that the optimum PENG from nanocomposite thin films can be a promising power source candidate for low-powered electronics. In addition, the stability of the optimum PZT-based PENG was evaluated by running it for an extended period. As shown in Figure 6c, the *V_OC_* signals remained consistent over 1000 s (2500 cycles at 2.5 Hz), indicating that the optimum PENG possesses good stability.

#### 3.4.2. Example of Application

**Figure 7:**
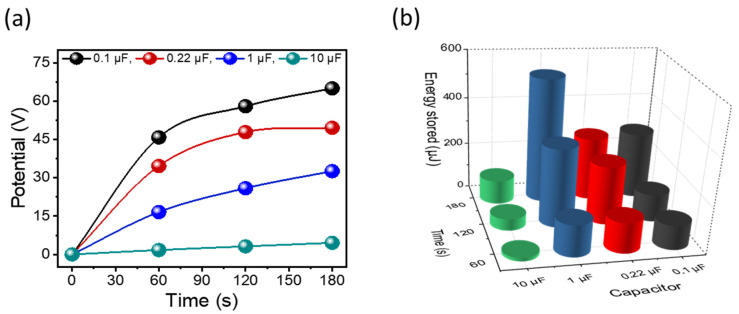
(**a**) Potential profiles and (**b**) energy storage values for the optimal PENG of thin film containing 4 wt% PZT with different capacitors.

The optimal PENG, incorporating 4 wt% PZT and referred to as the P-PZT-4 thin film, was anticipated to efficiently store electrical potential when paired with capacitors, achieving the highest power density for practical applications. To demonstrate its applicability, a simple circuit was constructed, consisting of a capacitor and four rectifiers necessary for converting the AC signals generated by the PENG into DC signals. Using this setup, electrical energy was successfully stored in capacitors with varying capacitances of 0.1, 0.22, 1, and 10 µF during bending–releasing cycles at 2.5 Hz. The stored energy (*E*) was calculated using the equation $E=\frac{1}{2}CV^{2}$ , where *C* represents the capacitance and *V* the potential [44]. The results, presented in Figure 7a,b, showed that the highest potential was achieved with a 0.1 µF capacitor, while the maximum stored electrical energy over 3 min (180 s) reached approximately 531.4 µJ with a 1 µF capacitor—sufficient to power multiple LEDs. These findings highlight the potential of the optimum PENG nanocomposite thin film containing 4 wt% PZT to power low-energy electronic devices, such as wearable electronics.

As detailed in Table 4, the performance of the optimal PENG in this study was compared with results from other PENGs based on similar materials, such as PVDF/PZT or P(VDF-TrFE)/PZT, as reported in the literature. Using a small amount of nanofiller, the current optimum PENG yielded a superior *V_OC_* value of 68.5 V and a fair *P*_2*D*_ value of 20.6 μW/cm^2^. However, this *P*_2*D*_ value is particularly impressive as it corresponds to a *P*_3*D*_ value of 8.6 W/cm^3^, which is a huge amount considering that a molecularly thin (24 nm thickness) film was used as the active material in this study.

This excellent PENG performance can be attributed to two main factors: First, the LS film deposition technique was able to provide highly ordered ultrathin nanocomposite films [49] with greater purity of the ferroelectric β-phase and strongly ordered structures [50], which enhanced crystallinity and thus overall piezoelectric properties. Second, the nanofiller type and content were properly optimized to maximize piezoelectric performance. Specifically, the thin film incorporating 4 wt% PZT, named P-PZT-4, exhibited the highest crystallinity, β-phase content, *d*_33_, and *V_OC_*. As seen in Tables S1–S4, all the results were complementary to one other in a systematic manner. Similarly, these behaviors of maximum piezoelectric (or triboelectric) properties at a specific filler content in nanocomposite films have been reported in the literature [15,25,47,48,51,52,53]. These results were explained in terms of electrical and/or dipole interactions between the filler phase and the polymer matrix. In addition, the current approach for PENGs based on nanocomposite thin films offers the advantage of a simple and cost-effective fabrication process, primarily originating from the LS technique used for film formation.

## 4. Conclusions

In summary, we investigated the influence of various nanofillers, including TiO_2_, ZnO, rGO, and PZT, on the performance of piezoelectric nanogenerator (PENG) thin films. These films were created by depositing molecularly thin layers of P(VDF-TrFE)/nanofiller NPs at the air/water interface onto an ITO-PET substrate. We fabricated several PENGs with different nanofiller compositions by arranging the coated thin films into a sandwich-like structure and evaluated their piezoelectric properties, such as *V_OC_* and *I_SC_*. The PENGwith 4 wt% PZT, i.e., P-PZT-4, exhibited the best performance, achieving a *V_OC_* of 68.5 V, a *d*_33_ of 78.2 pC/N, and a β-phase content of 97%. The PENGs’ *V_OC_* values followed this order: PZT (68.5V) > rGO (64.0V) > ZnO (50.9V) > TiO_2_ (48.1V). The two-dimensional power density for the optimal PENG of P-PZT-4 film was estimated to be 20.6 μW/cm^2^, which is about 2.3 times higher than those for the pristine-P(VDF-TrFE) film and about five times higher than a typical power density harvestable from solar energy. When integrated into a simple circuit with rectifiers and a capacitor, the optimal PENG demonstrated an impressive energy storage capacity of 531.4 μJ within 3 min. This PENG outperformed others in literature in terms of piezoelectric performance. As a result, this PENG, with improved performance due to optimized nanofiller type and content, shows great potential as a power source candidate for low-powered electronics.

## Figures and Tables

**Table 1:** Summary of crystallinity and β-phase content results.

Sample	Crystallinity (%)	β-Phase (%)	Sample	Crystallinity (%)	β-Phase (%)
P-TiO_2_-0	34	85	P-ZnO-0	34	85
P-TiO_2_-2	44	95	P-ZnO-2	50	96
P-TiO_2_-4	35	92	P-ZnO-4	45	94
P-TiO_2_-20	24	87	P-ZnO-20	40	90
P-TiO_2_-40	20	84	P-ZnO-40	32	84
Sample	Crystallinity (%)	β-phase (%)	Sample	Crystallinity (%)	β-phase (%)
P-rGO-0	34	85	P-PZT-0	34	85
P-rGO-2	42	92	P-PZT-2	40	91
P-rGO-4	56	97	P-PZT-4	52	97
P-rGO-20	32	92	P-PZT-20	36	86
P-rGO-40	39	90	P-PZT-40	29	82

**Table 2:** Summary of piezoelectric coefficient (*d*_33_) results.

Sample	*d*_33_ (pC/N)	Sample	*d*_33_ (pC/N)	Sample	*d*_33_ (pC/N)	Sample	*d*_33_ (pC/N)
P-TiO_2_-0	26.1	P-ZnO-0	26.1	P-rGO-0	26.1	P-PZT-0	26.1
P-TiO_2_-2	39.2	P-ZnO-2	58.1	P-rGO-2	36.3	P-PZT-2	35.8
P-TiO_2_-4	35.8	P-ZnO-4	46.8	P-rGO-4	69.4	P-PZT-4	78.2
P-TiO_2_-20	24.8	P-ZnO-20	45.2	P-rGO-20	39.6	P-PZT-20	30.4
P-TiO_2_-40	20.8	P-ZnO-40	31.4	P-rGO-40	36.2	P-PZT-40	22.2

**Table 3:** Comparison of current density and power density of PENGs comprising P-PZT-0 and P-PZT-4 films.

Sample	*V_OC_* (V)	*I_SC_* (μA)	*J_SC_* (μA/cm^2^)	*P*_2*D*_ (μW/cm^2^)	*P*_3*D*_ (W/cm^3^)
P-PZT-0	34.0	3.9	0.26	8.8	3.7
P-PZT-4	68.5	4.5	0.30	20.6	8.6

**Table 4:** Comparison of the optimum PENG performance in this study with those of other studies on PENGs based on PVDF/PZT or P(VDF-TrFE)/PZT reported in the literature.

Material	Filler Content (wt %)	Method	Sample Size (cm^2^)	*V_OC_* (V)	*P*_2*D*_ (μW/cm^2^)	Reference
PVDF/PZT	30	solution casting	2 × 0.9	55	36	[45]
PVDF/PZT	37	electrospinning	4 × 4	0.184	30.7	[46]
PVDF/PZT	10	hot press	6 × 6	1.7	0.196	[47]
P(VDF-TrFE)/m-PZT	10	solution casting	1 × 1	3.43	-	[48]
P(VDF-TrFE)/PZT NP	4	LS thin film	6 × 2.5	68.5	20.6	this work

## Data Availability

All the data are included within the article, and the raw data are available upon request.

## Supplementary Materials

The following supporting information can be downloaded at: https://www.mdpi.com/article/10.3390/nano15050403/s1.
