# Correction: Yaseen, H.M.A.; Park, S. Effect of Various Nanofillers on Piezoelectric Nanogenerator Performance of P(VDF-TrFE) Nanocomposite Thin Film. *Nanomaterials* 2025, *15*, 403

**DOI:** 10.3390/nano15231792

**Published:** 2025-11-28

**Authors:** Hafiz Muhammad Abid Yaseen, Sangkwon Park

**Affiliations:** Department of Chemical and Biochemical Engineering, Dongguk University, 30 Pildong-ro 1-gil, Jung-gu, Seoul 04620, Republic of Korea; dr.abidyaseen@gmail.com

In the published paper [1], there was an error regarding the order of the authorship. The authors submitted a formal request to the journal’s Editorial Office to correct this mistake, which occurred during submission. The updated authorship order is as follows:

Hafiz Muhammad Abid Yaseen and Sangkwon Park *
* corresponding author

The correspondence contact information of Hafiz Muhammad Abid Yaseen has also been updated. The authors state that the scientific conclusions are unaffected. The original publication has also been updated.
