# Supplementary material for: Effect of Various Nanofillers on Piezoelectric Nanogenerator Performance of P(VDF-TrFE) Nanocomposite Thin Film
Source: Nanomaterials (Basel). 2025 Mar 6;15(5):403. doi: 10.3390/nano15050403 (PMC11901528; doi:10.3390/nano15050403)
Supplement: Supplementary file 1 [file nanomaterials-15-00403-s001.zip › nanomaterials-3474520-supplementary.pdf]

Supplemental Information

Table S1. Summary of characterization and piezoelectric performance results for P(VDF-TrFE)/TiO_2 thin films

**Sample**	**Nanofiller Content (wt%)**	**Crystallinity (%)**	**β-phase (%)**	d_{33} **(pC/N)**	V_{OC} **(V)**
P-TiO_2-0	0	34	85	26.1	34.0
**P-TiO_2-2**	**2**	**44**	**95**	**39.2**	**48.1**
P-TiO_2-4	4	35	92	35.8	23.2
P-TiO_2-20	20	24	87	24.8	21.6
P-TiO_2-40	40	20	84	20.8	15.2

Table S2. Summary of characterization and piezoelectric performance results for P(VDF-TrFE)/ZnO thin films

**Sample**	**Nanofiller Content (wt%)**	**Crystallinity (%)**	**β-phase (%)**	d_{33} **(pC/N)**	V_{OC} **(V)**
P-ZnO-0	0	34	85	26.1	34.0
**P-ZnO-2**	**2**	**50**	**96**	**58.1**	**50.9**
P-ZnO-4	4	45	94	46.8	42.6
P-ZnO-20	20	40	90	45.2	35.4
P-ZnO-40	40	32	84	31.4	32.3

Table S3. Summary of characterization and piezoelectric performance results for P(VDF-TrFE)/rGO thin films

**Sample**	**Nanofiller Content (wt%)**	**Crystallinity (%)**	**β-phase (%)**	d_{33} **(pC/N)**	V_{OC} **(V)**
P-rGO-0	0	34	85	26.1	34.0
P-rGO-2	2	42	92	36.3	36.5
**P-rGO-4**	**4**	**56**	**97**	**69.4**	**64.4**
P-rGO-20	20	32	92	39.6	58.9
P-rGO-40	40	39	90	36.2	48.5

Table S4. Summary of characterization and piezoelectric performance results for P(VDF-TrFE)/rGO thin films

**Sample**	**Nanofiller Content (wt%)**	**Crystallinity (%)**	**β-phase (%)**	d_{33} **(pC/N)**	V_{OC} **(V)**
P-PZT-0	0	34	85	26.1	34.0
P-PZT-2	2	40	91	35.8	48.5
**P-PZT-4**	**4**	**52**	**97**	**78.2**	**68.5**
P-PZT-20	20	36	86	30.4	47.3
P-PZT-40	40	29	82	22.2	26.0
